# Age- and brain region-dependent α-synuclein oligomerization is attributed to alterations in intrinsic enzymes regulating α-synuclein phosphorylation in aging monkey brains

**DOI:** 10.18632/oncotarget.6445

**Published:** 2015-12-01

**Authors:** Min Chen, Weiwei Yang, Xin Li, Xuran Li, Peng Wang, Feng Yue, Hui Yang, Piu Chan, Shun Yu

**Affiliations:** ^1^ Department of Neurobiology, Xuanwu Hospital of Capital Medical University, Beijing, China; ^2^ Center of Parkinson's Disease, Beijing Institute for Brain Disorders, Beijing, China; ^3^ Beijing Key Laboratory for Parkinson's Disease, Beijing, China

**Keywords:** α-synuclein, polo-like kinase 2, protein phosphatase 2A, aging, brain, Gerotarget

## Abstract

We previously reported that the levels of α-syn oligomers, which play pivotal pathogenic roles in age-related Parkinson's disease (PD) and dementia with Lewy bodies, increase heterogeneously in the aging brain. Here, we show that exogenous α-syn incubated with brain extracts from older cynomolgus monkeys and in Lewy body pathology (LBP)-susceptible brain regions (striatum and hippocampus) forms higher amounts of phosphorylated and oligomeric α-syn than that in extracts from younger monkeys and LBP-insusceptible brain regions (cerebellum and occipital cortex). The increased α-syn phosphorylation and oligomerization in the brain extracts from older monkeys and in LBP-susceptible brain regions were associated with higher levels of polo-like kinase 2 (PLK2), an enzyme promoting α-syn phosphorylation, and lower activity of protein phosphatase 2A (PP2A), an enzyme inhibiting α-syn phosphorylation, in these brain extracts. Further, the extent of the age- and brain-dependent increase in α-syn phosphorylation and oligomerization was reduced by inhibition of PLK2 and activation of PP2A. Inversely, phosphorylated α-syn oligomers reduced the activity of PP2A and showed potent cytotoxicity. In addition, the activity of GCase and the levels of ceramide, a product of GCase shown to activate PP2A, were lower in brain extracts from older monkeys and in LBP-susceptible brain regions. Our results suggest a role for altered intrinsic metabolic enzymes in age- and brain region-dependent α-syn oligomerization in aging brains.

## INTRODUCTION

Aging is the most important risk factor for Parkinson's disease (PD) and dementia with Lewy bodies (DLB) [1, 2], which are pathologically characterized by the formation of fibrous protein inclusions known as Lewy bodies (LBs) and Lewy neurites (LNs), i.e. Lewy body pathology (LBP) [3, 4]. A major component of LBs and LNs are the fibrils of α-synuclein (α-syn), a small protein present in a soluble, monomeric form in normal neurons, which can aggregate into fibrils in pathological conditions [5, 6]. Although α-syn in LBs and LNs is fibrotic, increasing evidence suggests that it is the small α-syn aggregates (oligomers and protofibrils) rather than the α-syn fibrils that are toxic to neurons [7-10]. Oligomeric α-syn can not only induce neuronal death [7-13], but also cause synaptic dysfunctions leading to cognitive impairment in PD and DLB [14-17]. Thus, the accumulation of oligomeric α-syn in the brain may increase the vulnerability of neurons to degeneration.

We previously reported that aging in the brain is associated with an increase in α-syn oligomers, with the effect differing between certain brain regions [18]. However, the intrinsic factors that lead to the age- and brain region-dependent accumulation of α-syn oligomers remains elusive. It has been shown that around 90% of the α-syn in LBs are serine 129 phosphorylated α-syn (pS129 α-syn) [19]. This raises the possibility that α-syn phosphorylation is the main cause for its aggregation. The role of phosphorylation in α-syn aggregation was later confirmed by studies performed in cell lines, which associated phosphorylated α-syn with increased formation of soluble oligomers [20-22]. Two enzymes appear to play a major role in the regulation of α-syn phosphorylation. One is polo-like kinase 2 (PLK2), which has been shown to promote α-syn phosphorylation, especially at serine 129 [23-25]. Increased expression of PLK2 was observed in the brains of patients with Alzheimer's disease and LB disease [26]. The other one is protein phosphatase 2A (PP2A), which has been found to facilitate α-syn dephosphorylation [28, 29]. It has been reported that in DLB and α-syn triplication brains, which contain robust α-syn aggregation with high levels of serine 129 phosphorylation, PP2A activity was attenuated by 50% [30], indicating a potential link between PP2A activity and α-syn phosphorylation and aggregation. However, none of the previous studies have investigated how PLK2 and PP2A change in the aging brain and whether their changes are associated with the age- and brain region-dependent α-syn oligomerization.

Mutations in the *GBA* (glucosidase, beta, acid) gene encoding β-glucocerebrosidase (GCase), which cause Gaucher disease [31], are recognized risk factors for PD [32, 33]. GCase is a lysosomal enzyme that hydrolyzes glucosylceramide (GlcCer) into glucose and ceramide [31]. Mutations to the *GBA* gene can lead to the inhibition of the lysosomal function of GCase and the accumulation of GlcCer, which promotes α-syn oligomerization by stabilizing soluble oligomeric intermediates [34]. The accumulation of oligomeric α-syn can alter the activity of GCase by modulating its transport from the endoplasmic reticulum to the lysosome [34-36]. Additionally, it may also enable an increase in α-syn phosphorylation by reducing the activity of PP2A via decreased production of ceramide, an activator of PP2A [37]. While there is evidence suggesting an inverse relationship between the reduced activity of GCase and increased levels of ceramide in the brains of patients with PD [38], variations in GCase in the aging brain and their potential links to the age- and brain region-dependent α-syn oligomerization remain unknown.

In the present study, we examined α-syn oligomerization and phosphorylation by incubating recombinant human α-syn in extracts isolated from brain regions (the striatum and hippocampus) relatively susceptible to LBP and those (the cerebellum and occipital cortex) relatively insusceptible to LBP [3, 4, 39] of cynomolgus monkeys of varying age. We analyzed how differential alterations of PLK2, PP2A, GCase, and ceramide in the aging brain influence α-syn phosphorylation, as well as corresponding age- and brain region-dependent α-syn oligomerization.

## RESULTS

### Depletion of endogenous α-syn by anti-α-syn antibody

**Figure 1:**
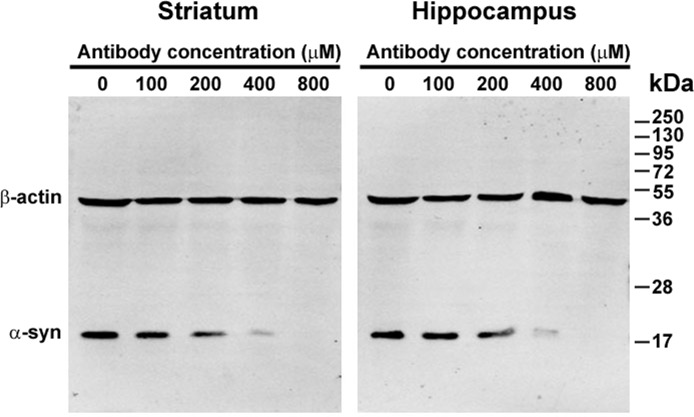
Depletion of endogenous α-synuclein (α-syn) by anti-α-syn antibody Extracts of the striatum and hippocampus were incubated with different concentrations of the 3D5 mouse monoclonal antibody conjugated to Protein G. The antibody-Protein G-endogenous α-syn complex was removed by centrifugation. The supernatants were then examined by western blotting. With an increase in the 3D5 antibody concentration, the levels of endogenous α-syn in the supernatants decreased and disappeared.

Because the presence of endogenous α-syn may interfere with the phosphorylation and oligomerization of exogenous α-syn in brain extracts, the endogenous α-syn was first depleted using an 3D5 anti-α-syn antibody recognizing a specific sequence of human and cynomolgus monkey α-syn [40]. To find the minimal antibody concentration needed for complete depletion of the endogenous α-syn, brain extracts from the striatum and hippocampus with a protein concentration of 1 mg/ml were incubated with different concentrations of the anti-α-syn antibody conjugated to Protein G for 24 h at 37°C. The antibody-Protein G-endogenous α-syn complex was removed by centrifugation and the supernatants were then examined by western blotting. The amount of endogenous α-syn gradually decreased with an increase in antibody concentration. Complete depletion of the endogenous α-syn was achieved when the antibody concentration reached 800 μM (Figure 1). Because the striatum and hippocampus contain higher concentrations of endogenous α-syn, we reasoned that incubation with 800 μM of anti-α-syn antibody for 24 h was sufficient to deplete the endogenous α-syn of extracts from other brain regions. Therefore, in subsequent experiments, 800 μM of the anti-α-syn antibody was used to deplete the endogenous α-syn in brain extracts.

### Brain extracts promote α-syn oligomerization and phosphorylation

**Figure 2:**
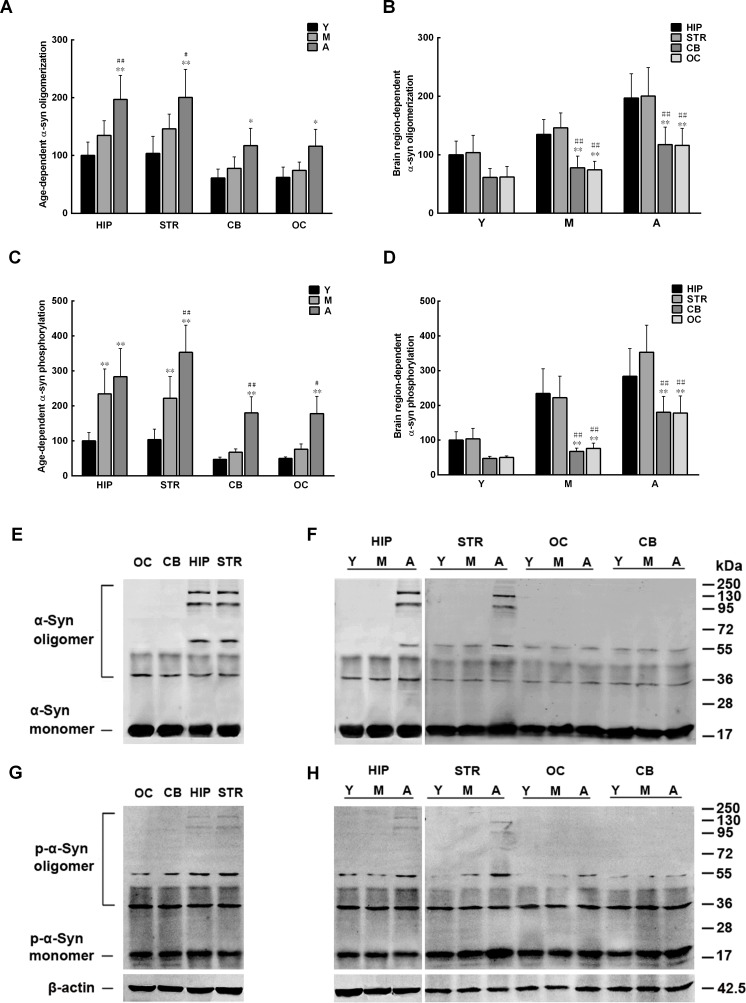
Age-and brain region-dependent alterations in α-synuclein (α-syn) oligomerization and phosphorylation Recombinant α-syn was added to brain extracts depleted of endogenous α-syn, and incubated at 37°C for 48 h with constant shaking. The amounts of oligomeric and pS129 α-syn formed in the extracts were measured by ELISA and Western blot. A/E. α-syn oligomerization (%)increases with age. B/F. α-syn oligomerization differs between brain regions. C/G. α-syn phosphorylation (%) increases with age. D/H. α-syn phosphorylation differs between brain regions. Data are expressed as the mean ± SD. Tukey's multiple comparisons test after two-way ANOVA, A/C: *P < 0.05, **P < 0.01, vs. young age group in the same brain region (n = 5); ^#^*P* < 0.05, ^##^*P* < 0.01 vs. middle age group in the same brain region (n = 5); B/D: *P < 0.05, **P < 0.01, vs. the same age group in hippocampus (n = 5); ^#^*P* < 0.05, ^##^*P* < 0.01 vs. the same age group in striatum (n = 5). STR: striatum; HIP: hippocampus; CB: cerebellum; OC: occipital cortex. Y: young age group; M: middle age group; A: aged group.

We previously demonstrated that the levels of α-syn oligomers in the monkey brain differ between brain regions and increase with age [18]. We speculate that the discrepancy between brain regions and the age-dependent elevation of α-syn oligomer levels can be ascribed to intrinsic metabolic factors in the brain. To test this hypothesis, the total protein concentration of extracts from several brain regions of cynomolgus monkeys of different ages was first adjusted to 1 mg in 1 ml of PBS. Then, the extracts, after depleted of endogenous α-syn, were incubated at 37°C for 48 h with continuous shaking (650 rpm) with recombinant human α-syn (100 μM) to observe its phosphorylation and oligomerization. Both ELISA and western blot showed that the recombinant α-syn incubated in the extracts from the hippocampus and striatum formed more oligomers than that incubated in the extracts from the cerebellum and occipital cortex. In addition, α-syn oligomerization in extracts from all brain regions increased dramatically with age (Figure 2A, 2B, 2E, 2F). Similar to α-syn oligomerization, the amounts of pS129 α-syn formed in extracts from the hippocampus and striatum were also greater than those in extracts from the cerebellum and occipital cortex, which also increased significantly with age (Figure 2C, 2D, 2G, 2H). The above results suggest a potential link between altered α-syn phosphorylation and age- and brain region-dependent α-syn oligomerization.

### Age- and brain region-dependent alterations in PLK2 and PP2A

**Figure 3:**
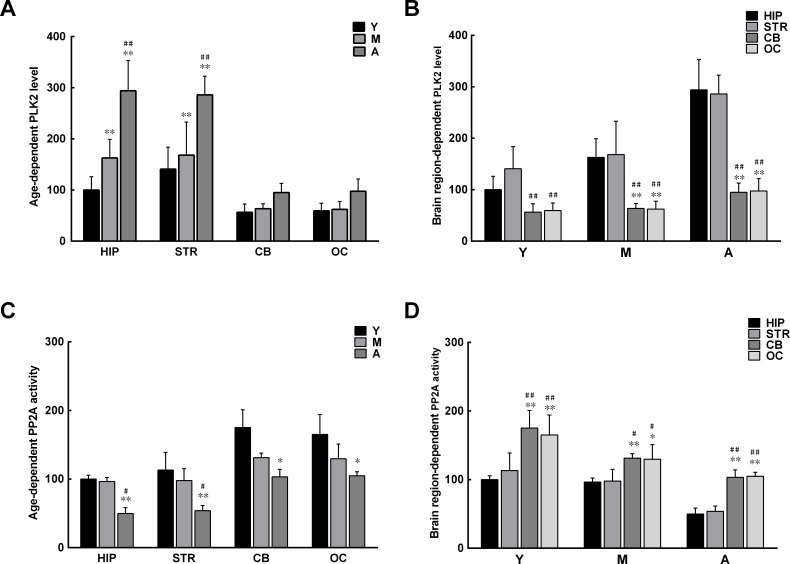
Age- and brain region-dependent alterations in polo-like kinase 2 (PLK2) expression and protein phosphatase 2A (PP2A) activity **A**. Levels of PLK2 in different brain regions increase with age. **B**. Levels of PLK2 differ between brain regions. **C**. PP2A activity in different brain regions decreases with age. **D**. PP2A activity differs between brain regions. Data are expressed as the mean ± SD. Tukey's multiple comparisons test after two way ANOVA, A/C: *P < 0.05, **P < 0.01, vs. young age group in the same brain region (n = 5); ^#^*P* < 0.05, ^##^*P* < 0.01 vs. middle age group in the same brain region (n = 5); B/D: *P < 0.05, **P < 0.01, vs. the same age group in hippocampus (n = 5); ^#^*P* < 0.05, ^##^*P* < 0.01 vs. the same age group in striatum (n = 5). STR: striatum; HIP: hippocampus; CB: cerebellum; OC: occipital cortex. Y: young age group; M: middle age group; A: aged group.

Since α-syn phosphorylation in brain extracts increases with age, it is possible that the activity of enzymes regulating α-syn phosphorylation is different in extracts isolated from monkey brains of different ages and from different regions of the brain. Two enzymes were examined: PLK2, the major enzyme shown to promote α-syn phosphorylation [23-25], and PP2A, the key enzyme found to dephosphorylate α-syn [28, 29]. An evidently higher level of PLK2 was observed in the extracts from the hippocampus and striatum compared with those from the cerebellum and occipital cortex, with a corresponding increase with age (Figure 3A, 3B). In contrast, the activity of PP2A was significantly lower in the extracts from the hippocampus and striatum than in those from the cerebellum and occipital cortex; the activity also decreased significantly with age (Figure 3C, 3D). The results indicate that the age- and brain region-dependent alterations in PLK2 and PP2A are associated with age- and brain region-dependent α-syn phosphorylation and oligomerization.

### Inhibition of PLK2 and activation of PP2A reduce α-syn phosphorylation and oligomerization

**Figure 4:**
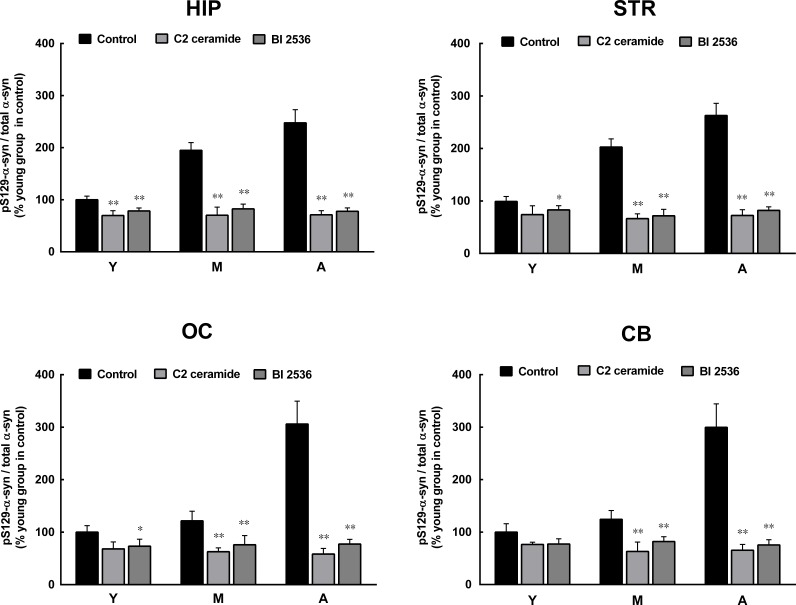
Effects of a protein phosphatase 2A (PP2A) activator and polo-like kinase 2 (PLK2) inhibitor on α-syn phosphorylation C2 ceramide (5 μM, 8 h), an activator of PP2A, and BI 2536 (1 μM, 24 h), an inhibitor of PLK2, significantly reduced the formation of pS129 α-syn in extracts from all brain regions. Data are expressed as the mean ± SD. Tukey's multiple comparisons test after two way ANOVA, *P < 0.05, **P < 0.01 vs. the same age group in hippocampus (n = 5). STR: striatum; HIP: hippocampus; CB: cerebellum; OC: occipital cortex. Y: young age group; M: middle age group; A: aged group.

**Figure 5:**
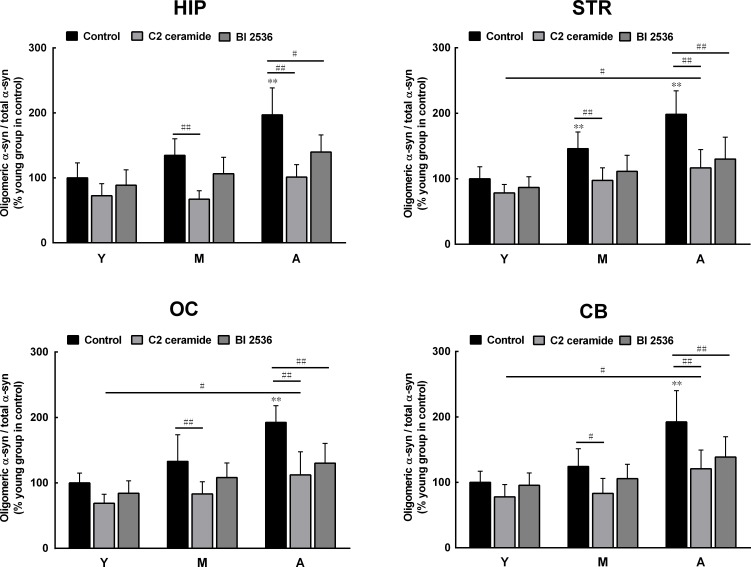
Effects of a protein phosphatase 2A (PP2A) activator and polo-like kinase 2 (PLK2) inhibitor on α-syn oligomerization C2 ceramide (5 μM, 8 h), an activator of PP2A, and BI 2536 (1 μM, 24 h), an inhibitor of PLK2, significantly reduced the formation of oligomeric α-syn in extracts from all brain regions. Data are expressed as the mean ± SD. Tukey's multiple comparisons test after two way ANOVA, **P < 0.01 vs. the young age group in hippocampus (n = 5); ^#^ *P* < 0.05, ^##^ *P* < 0.01 vs. as indicated (n = 5). STR: striatum; HIP: hippocampus; CB: cerebellum; OC: occipital cortex. Y: young age group; M: middle age group; A: aged group.

To confirm the roles of PLK2 and PP2A in age-dependent α-syn phosphorylation and oligomerization, we observed the effects of PLK2 inhibition and PP2A activation on α-syn phosphorylation and oligomerization. The presence of PLK2 inhibitor BI 2536 and PP2A activator C2 ceramide significantly reduced α-syn phosphorylation in extracts from all regions of the brain and in monkey brains of all ages. The age-dependent increase in α-syn phosphorylation was abolished by BI 2536 and C2 ceramide (Figure 4). In addition, BI 2536 and C2 ceramide also reduced the age-dependent α-syn oligomerization (Figure 5), indicating that the age-dependent α-syn oligomerization is associated with the age-dependent α-syn phosphorylation.

### Age-and brain region-dependent reduction in levels of ceramide

**Figure 6:**
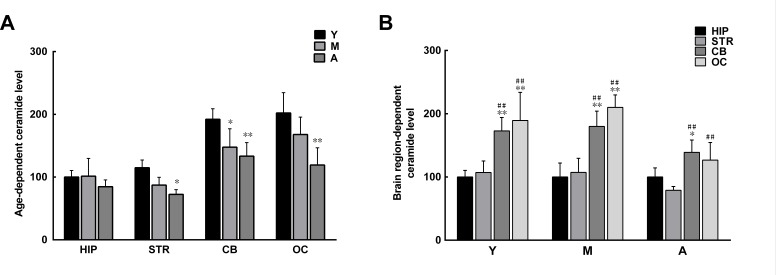
Age- and brain region-dependent differences in ceramide levels **A**. Ceramide levels tended to decrease with age in all brain regions, with significant reduction observed in the striatum, cerebellum, and occipital cortex. **B**. In the same age group, the levels of ceramide in the cerebellum and occipital cortex were significantly higher than those in the striatum and hippocampus. Data are expressed as the mean ± SD. Tukey's multiple comparisons test after two way ANOVA, A: *P < 0.05, **P < 0.01, vs. young age group in the same brain region (n = 5); B: *P < 0.05, **P < 0.01, vs. the same age group in hippocampus (n = 5); ^#^ *P* < 0.05, ^##^ *P* < 0.01 vs. the same age group in striatum (n = 5). STR: striatum; HIP: hippocampus; CB: cerebellum; OC: occipital cortex. Y: young age group; M: middle age group; A: aged group.

The above results show that the synthetic C2 ceramide can reduce α-syn phosphorylation and oligomerization in brain extracts. Since ceramide has been shown to activate PP2A [37], it is likely that in those brain regions with reduced PP2A activity there is a reduction in levels of naturally occurring ceramide. We measured ceramide in brain extracts and found that the levels of ceramide in the striatum and hippocampus were much lower than those in the cerebellum and occipital cortex. In addition, the levels of ceramide in extracts from all brain regions underwent an age-dependent reduction (Figure 6). The alterations in ceramide levels paralleled those in PP2A activity, indicating a link between ceramide and PP2A.

### Age- and brain region-dependent decrease in GCase activity

**Figure 7:**
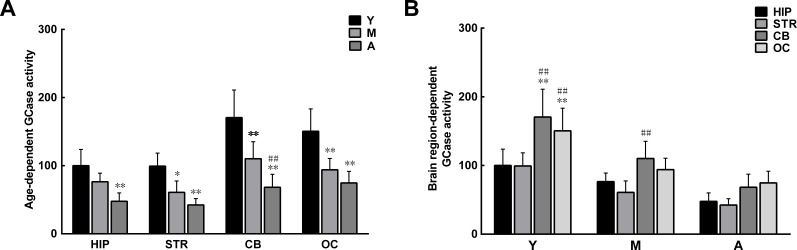
Age- and brain region-dependent differences in glucocerebrosidase (GCase) activity **A**. The GCase activity tended to decrease with age in all brain regions. **B**. In the same age group, GCase activity differed between brain regions, with relatively higher activity observed in the striatum and hippocampus. Data are expressed as the mean ± SD. Tukey's multiple comparisons test after two way ANOVA, A: *P < 0.05, **P < 0.01, vs. young age group in the same brain region (n = 5); ^##^*P* < 0.01, vs. middle age group in the same brain region (n = 5); B: *P < 0.05, **P < 0.01, vs. the same age group in hippocampus (n = 5); ^#^*P* < 0.05, ^##^*P* < 0.01 vs. the same age group in striatum (n = 5). STR: striatum; HIP: hippocampus; CB: cerebellum; OC: occipital cortex. Y: young age group; M: middle age group; A: aged group.

The reduction in levels of ceramide with increasing age may result from impaired GCase activity in the aging brain, since ceramide is a hydrolytic product of glucosylceramide catalyzed by GCase [31]. As predicted, GCase activity was discrepant between extracts from different brain regions. The GCase activity in extracts from the hippocampus and striatum was lower than that in extracts from the cerebellum and occipital cortex. With increasing age, GCase activity further decreased in all brain extracts (Figure 7).

### α-Syn oligomers reduce PP2A activity

**Figure 8:**
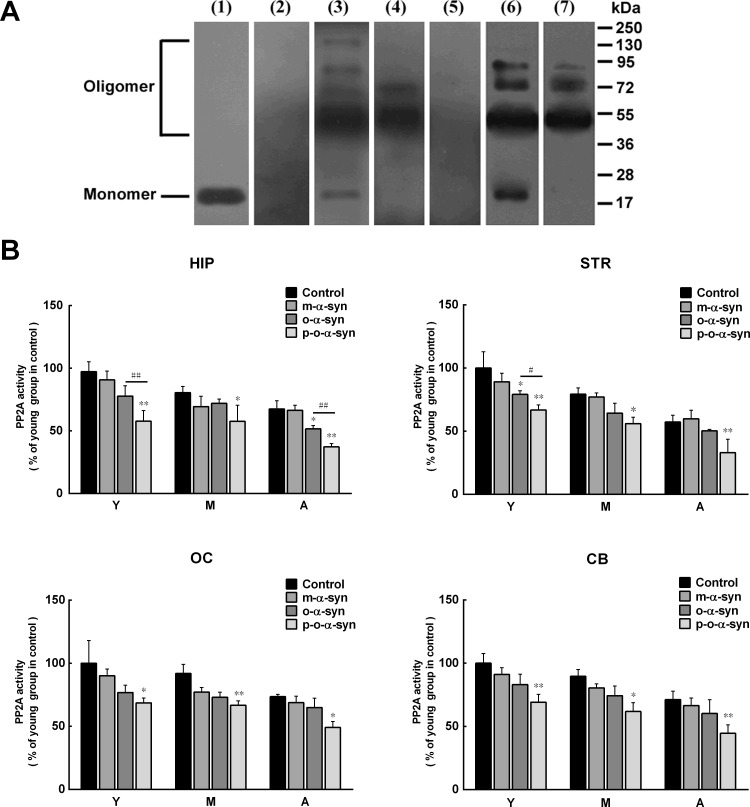
Effect of phosphorylated and non-phosphorylated oligomeric α-syn on PP2A activity **A**. Western blot analysis of α-syn. The brain extract from the striatum was detected by 3D5 mouse monoclonal antibody (lane 1) against an epitope ranging from 115 to 121 residues of human α-syn [41], which revealed an 18 kDa endogenous α-syn. The endogenous α-syn was disappeared after the extract was pre-absorbed by overdose of anti-α-syn antibody (lane 2). Recombinant α-syn incubated in PBS was detected by the 3D5 antibody, which revealed monomeric and various sizes of oligomeric α-syn (lane 3). After purification, the monomeric α-syn was removed (lane 4). The oligomeric α-syn formed in PBS was not detected by the anti-pS129 α-syn antibody (lane 5). The recombinant α-syn incubated in the brain extract from the striatum was detected by the anti-pS129 α-syn antibody before (lane 6) and after (lane 7) purification. **B**. The purified monomeric α-syn as well as phosphorylated and non-phosphorylated oligomeric α-syn was added to the brain extracts to observe their effects on PP2A activity. No significant reduction of PP2A activity was observed in monomeric α-syn-treated brain extracts. In contrast, in brain extracts treated with oligomeric α-syn (whether or not it is phosphorylated), significant reduction of PP2A activity was detected. In comparison with the non-phosphorylated oligomeric α-syn, the phosphorylated oligomeric α-syn exhibited more potent effect on PP2A activity. Data are expressed as the mean ± SD. Tukey's multiple comparisons test after two way ANOVA, *P < 0.05, **P < 0.01, vs. the control groups; (n = 5); ^#^ *P* < 0.05, ^##^*P* < 0.01 vs. the non-phosphorylated oligomeric α-syn group (n = 5).

It has been reported that PP2A activity is reduced in DLB and α-syn triplication brains, which contain robust α-syn aggregation with high levels of serine 129 phosphorylation [27]. Given this, it is possible that reduced PP2A activity in the brain extracts from aged monkeys and susceptible brain regions may result from increased phosphorylated α-syn aggregates, in addition to decreased ceramide levels. To demonstrate this, recombinant human α-syn was incubated with the extract from the striatum of an old monkey, and the phosphorylated α-syn oligomers were isolated and purified. As a control, non-phosphorylated α-syn oligomers were prepared and purified by incubating recombinant human α-syn in PBS. Western blot analysis showed that the oligomeric α-syn prepared in PBS was recognized by the 3D5 anti-non-phosphorylated α-syn antibody but not by an antibody targeting pS129 α-syn. In contrast, the oligomeric α-syn prepared in brain extracts was recognized by not only the 3D5 antibody but also the antibody against pS129 α-syn. The molecular size of the α-syn oligomers formed in PBS and brain extracts mainly ranged from 54-90 kDa, which corresponded to trimers, tetramers, and pentamers (Figure 8A). We added the monomeric and oligomeric α-syn (either phosphorylated or non-phosphorylated) to the brain extracts depleted of the endogenous α-syn and incubated them for 24 h. We then measured the PP2A activity in the extracts. In brain extracts incubated with monomeric α-syn, the PP2A activity was slightly reduced, but this reduction was not statistically significant. However, in brain extracts incubated with either phosphorylated or non-phosphorylated oligomeric α-syn, the reduction of PP2A activity was significant, suggesting a potential role for oligomeric α-syn in inhibiting PP2A activity. Compared with the non-phosphorylated oligomeric α-syn, the phosphorylated oligomeric α-syn exhibited more potent effect on PP2A activity (Figure 8B).

### Cell toxicity of phosphorylated oligomeric α-syn

**Figure 9:**
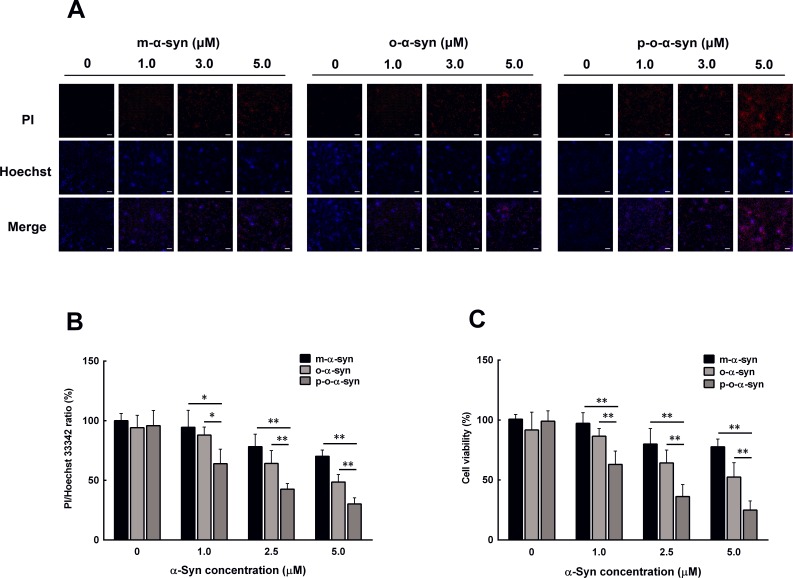
Effect of phosphorylated oligomeric α-syn on dopaminergic neuronal cells Non-phosphorylated and phosphorylatedα-syn oligomers were prepared by incubating recombinant human α-syn with either PBS or the brain extract from the hippocampus of an aged monkey. After purification, different concentrations of the α-syn monomer and non-phosphorylated and phosphorylatedα-syn oligomers were added to the culture medium of MES23.5 dopaminergic cells. 24 h later, the cell death rate and cell viability were evaluated with PI/Hoechst staining and MTT assay. **A**. Photomicrographs showing the cells stained by PI/Hoechst. In both the monomeric and oligomeric α-syn-treated cells, the counts of PI-positive nuclei (red color) increased with an increase in α-syn concentration. This increase was most prominent in the phosphorylated oligomeric α-syn-treated cells. Scale bar = 10 μM **B**. Statistical results showing the ratios of PI/Hoechst 33342-positive cells. **C**. MTT assay of the cell viability. Data are expressed as the mean ± SD. Tukey's multiple comparisons test after two way ANOVA, *P < 0.05, **P < 0.01 vs. the phosphorylated oligomeric α-syn group (n = 5).

Since aging is accompanied by increased accumulation of phosphorylated α-syn oligomers, it is worth knowing if this form of α-syn is toxic to neuronal cells. To this end, non-phosphorylated and phosphorylated α-syn oligomers were added to the culture medium of MES23.5 dopaminergic cells. As a control, purified α-syn monomers were used to treat other groups of cells. PI/Hoechst staining showed that the cell death rate was significantly increased when 1 μM of the non-phosphorylated and phosphorylated α-syn oligomers were used to treat the cells. In contrast, the same concentration of the monomeric α-syn did not induce significant change in cell death rate. When the α-syn concentration was further increased, both the monomers and oligomers induced significant elevation in cell death rate. The phosphorylated α-syn oligomers showed the most potent effect on cell death when compared with the monomers and non-phosphorylated α-syn oligomers (Figure 9A, 9B). Similar results were also obtained by MTT assay, which showed the most prominent reduction in cell viability in the phosphorylated α-syn oligomer-treated cells when compared with those in the monomer and non-phosphorylated α-syn oligomer-treated cells (Figure 9C).

## DISCUSSION

In the present study, we found that exogenous α-syn incubated with the brain extracts of cynomolgus monkeys can aggregate into oligomers. The amount of α-syn oligomers formed in the extracts depended on the brain region and the age of monkeys from which the brain extracts were obtained. More α-syn oligomers were formed in the extracts from the striatum and hippocampus (LBP-susceptible regions) than in those from the cerebellum and occipital cortex (LBP-insusceptible regions) [3, 4, 39]. In addition, α-syn oligomerization in brain extracts significantly increased with an increase in the age of the animal. The findings of age- and brain region-dependent α-syn oligomerization in brain extracts were identical to our previous reports that higher levels of α-syn oligomers are observed in PD-susceptible brain regions and in aged monkeys [18]. Since α-syn phosphorylation, especially at serine 129, has been shown to promote α-syn aggregation [20-22], we also measured α-syn phosphorylation in the brain extracts. Similar to the trend of the change in α-syn oligomerization, α-syn phosphorylation in extracts from the striatum and hippocampus was also higher than in those from the cerebellum and occipital cortex, and increased with age. The parallel alterations in α-syn phosphorylation and oligomerization suggest that the altered α-syn phosphorylation is the major reason for the age- and brain region-dependent α-syn oligomerization. However, other potential mechanisms should not be ruled out.

It has been shown that α-syn phosphorylation is regulated by two main enzymes. One is PLK2, a kinase that is shown to promote α-syn phosphorylation at serine 129 [23-25] and is highly expressed in the brain of patients with Alzheimer's disease and LB disease [26]. The other one is PP2A, a phosphatase shown to dephosphorylate α-syn [28, 29], whose activity is reduced in DLB and α-syn triplication brains, which contain robust α-syn aggregation with high levels of serine 129 phosphorylation [27]. Therefore, the age- and brain region-dependent alterations in α-syn phosphorylation may result from the differential expression and activity of PLK2 and PP2A in the aging brain. To demonstrate this possibility, we measured the levels of PLK2 and the activity of PP2A in the brain extracts. The results showed that the levels of PLK2 were higher in brain extracts from the hippocampus and striatum than in those from the cerebellum and occipital cortex. PLK2 levels were also higher in the extracts from older subjects. In contrast, PP2A activity was lower in brain extracts from the hippocampus and striatum than in those from the cerebellum and occipital cortex. PP2A activity reduced with an increase in age. These patterns of alterations in PLK2 and PP2A favor α-syn phosphorylation and oligomerization in the aging brain. To further confirm the roles of PLK2 and PP2A in the aging brain, we observed the effects of PLK2 inhibition and PP2A activation on α-syn phosphorylation and oligomerization. BI 2536, a PLK2 inhibitor, and C2 ceramide, a PP2A activator, significantly reduced α-syn phosphorylation in brain extracts from all brain regions and in monkeys of all ages. The results using PLK2 inhibitor and PP2A activator consolidated the conjecture that the increased α-syn phosphorylation in aged monkey brains results from the altered PLK2 and PP2A activity.

Due to the important role of PP2A in age-dependent α-syn oligomerization, we set out to investigate the potential mechanism for reduced PP2A activity in the aging brain. Previous studies have revealed a bidirectional positive feedback loop between α-syn and GCase [34]. On one hand, loss of GCase activity leads to the accumulation of GlcCer, which stabilizes α-syn oligomers; on the other hand, α-syn accumulation blocks the transport of GCase from the endoplasmic reticulum to the lysosome, resulting in GCase depletion in the lysosome. In the brains of patients with PD, there is a reduction in GCase activity and ceramide levels [38]. Because ceramide is a hydrolytic product of GlcCer when it is catalyzed by GCase, and is an activator of PP2A [37], its reduction due to impaired GCase activity would attenuate PP2A activity. In accordance with the changes in PP2A activity, the levels of ceramide and activity of GCase were lower in extracts from the striatum and hippocampus than in those from the cerebellum and occipital cortex. A similar reduction was observed with increasing age. Thus, the reduction of ceramide levels in the aging brain is one of the mechanisms that can result in reduced PP2A activity. In addition, a previous study suggested that the reduced PP2A activity in brains showing DLB and α-syn triplication may be caused by the attenuated stimulatory effect of α-syn aggregates on PP2A activity [27]. However, our present results, obtained by directly incubating phosphorylated α-syn oligomers with brain extracts, indicate that phosphorylated α-syn oligomers may decrease PP2A activity, although the detailed mechanism by which this effect occurs remains to be investigated.

Since the phosphorylated α-syn oligomers accumulate in the aging brain, we investigated the potential toxic role of this form of α-syn in cultured dopaminergic cells. We found that the phosphorylated α-syn oligomers induced the most remarkable elevation in cell death rate and reduction in cell viability when compared with α-syn. This indicates that the phosphorylated α-syn oligomers have a potential neurotoxic effect, although in the normal aging brain they may not induce obvious neurodegeneration, possibly due to the lower levels. However, the accumulation of the phosphorylated α-syn oligomers in the aging brain, especially in those LBP-susceptible brain regions such as the striatum and hippocampus, will increase the vulnerability to LBP and neurodegeneration.

## CONCLUSIONS

The present study demonstrates that α-syn phosphorylation and oligomerization in brain extracts differ between brain regions and increase with age. These changes are associated with alterations in intrinsic enzymes (PP2A, PLK2, and GCase) that regulate α-syn phosphorylation and oligomerization. The age- and brain region-dependent α-syn oligomerization in aging brains can explain why some brain regions, such as the striatum and hippocampus, are easily affected by LBP and preferentially degenerated in PD and DLB.

## MATERIALS AND METHODS

### Animals

Cynomolgus monkeys (*Macaca fascicularis*; n = 15) with detailed individual birth records and quarantine certificates were purchased from a local nonhuman primate breeder (Grandforest Co., Guangxi, China). All animals were healthy and without physical impairments. The animals were acclimated to the laboratory environment for at least 2 months prior to dissection and were divided into three age groups: young (range: 3–4 years; n = 5), middle age (range: 10–12 years; n = 5), and aged (range ≥15 years; n = 5). Animals were housed in a primate facility (Wincon TheraCells Biotechnologies Co., Ltd., Nanning, Guangxi, China) accredited by the Association for Assessment and Accreditation of Laboratory Animal Care under a 12:12 h light/dark cycle with free access to an uninterrupted reverse osmosis water supply. Food was available twice daily and supplemented with fresh fruit and vegetables. The experimental protocol was approved by the Institutional Animal Care and Use Committee of Wincon TheraCells Biotechnologies (permit no.WD-0312010). Investigation has been conducted in accordance with the ethical standards and according to the Declaration of Helsinki and according to national and international guidelines and has been approved by the local animal care and use committee.

All animal experiments were carried out in accordance with the National Institutes of Health (NIH) Guide for the Care and Use of Laboratory Animals (NIH publication no. 85-23, revised 1996).

### Dissection of monkey brain tissues

Monkeys were anaesthetized with 7-10 mg ketamine and were perfused with 0.01 mM phosphate buffered saline (pH 7.4) before the brains were removed from the skull. The brain tissues were dissected, snap-frozen in liquid nitrogen and stored at −80°C until use.

### Preparation of brain extracts

Tissues from different brain regions were lysed in a lysis buffer containing 50 mM Tris–HCl, pH 7.4, 150 mM NaCl, 1 mM EDTA. The lysate was centrifuged at 12,000 × g for 20 min at 4°C and the supernatant was aliquoted and stored at −80°C until use. Protein concentration was determined using the bicinchoninic acid (BCA) Protein Assay Kit (Pierce/Thermo Scientific, Waltham, MA, USA) according to the manufacturer's instructions.

### Depletion of endogenous α-syn in brain extracts

To deplete the endogenous α-syn, brain extracts containing 50 μg of protein were incubated with different concentrations of an anti-α-syn antibody at 4°C for 24 h. The antibody-α-syn complex was removed by using Protein G sepharose Fast Flow (P3296; Sigma-Aldrich, St. Louis, MO, USA). Complete depletion of the endogenous α-syn was examined by western blot.

### Preparation of oligomeric and phosphorylated α-syns

Oligomeric and phosphorylated α-syns were prepared as the standards for quantitative measurement of oligomeric α-syn and pS129 α-syn with Enzyme-linked immunosorbent assays (ELISAs). To prepare oligomeric α-syn standard, recombinant human α-syn was solubilized in sterile phosphate-buffered saline (PBS; pH 7.0) to a final concentration of 100 μM. An Eppendorf tube containing 100 μM of α-syn solution was sealed with parafilm and incubated at 37°C for 7 days with continuous shaking (650 rpm) on an Eppendorf Thermomixer Comfort (Z605271; Sigma-Aldrich, St. Louis, MO, USA). The incubated solution was further subjected to SDS-PAGE to isolate the α-syn oligomers from the dimers and monomers. The α-syn oligomers were purified from the gel using a Micro Protein Recovery Kit (Sangon, Biotech, Shanghai, China) according to the protocol provided. They were then aliquoted and stored at −80°C until use.

pS129 α-syn was prepared from recombinant human α-syn as previously described [41]. Purified α-syn was first incubated with casein kinase II (New England Biolabs, Ipswich, MA, USA) and the resultant pS129 α-syn was purified by anion-exchange chromatography and verified by immunoblotting with an antibody against the pS129 α-syn (Epitomics, Burlingame, CA, USA) combined with mass spectrometry. The pS129 α-syn was concentrated by ammonium sulfate precipitation.

To study the effect of phosphorylated α-syn oligomers on PP2A activity in brain extracts, 100 μM of recombinant α-syn was incubated at 37°C for 2 days with continuous shaking (650 rpm) with the extract isolated from the striatum of an aged monkey. The phosphorylated α-syn oligomers formed in the brain extract was purified using the method for purification of α-syn oligomers.

### ELISAs for oligomeric α-syn and pS129 α-syn

The oligomeric α-syn concentration in brain tissues and cell lysates was measured by ELISA [18, 42] using non-biotinylated and biotinylated 3D5 mouse monoclonal antibodies [40] for capture and detection, respectively. After completion of the immunoreaction, the contents of each well of the ELISA plate were incubated with 100 μl of ExtrAvidin Alkaline Phosphatase (E-2636; Sigma-Aldrich, St. Louis, MO, USA) diluted at a ratio of 1: 20,000 in blocking buffer, and then reacted with the enzyme substrate p-nitrophenyl phosphate (N1891; Sigma-Aldrich, St. Louis, MO, USA). The reaction was allowed to proceed for 30 min at room temperature, after which the absorbance was read at 405 nm using a Multiskan MK3 microplate reader (Thermo Scientific, UT, USA).

To detect pS129 α-syn, 0.1 μg/ml of anti-pS129 α-syn polyclonal antibody (Santa Cruz Biotechnology, Santa Cruz, CA, USA) in coating buffer was used for capture. The remaining steps were the same as for the detection of oligomeric α-syn by ELISA.

### PP2A activity assay

PP2A activity in the brain homogenates was measured as previously described [43] using a PP2A Colorimetric Assay Kit (GenMed Scientific Inc., Arlington, MA, USA). The protein concentration in the supernatant was determined by the Bradford assay (GMS 30030.1; GenMed Scientific Inc.) and was normalized to 5 mg/ml.

### GCase activity assay

GCase activity was determined using the QuantiChrom β-Glucosidase Assay kit (DBGD-100; BioAssay Systems Inc., Hayward, CA, USA). Distilled water (20 μl) was added to two wells of a clear-bottom 96-well plate; 200 μl of either distilled water or calibrator were then added to the wells to obtain a total volume of 220 μl. Samples (20 μl) were loaded in the other wells, and 200 μl of working reagent was added to each sample to obtain a final reaction volume of 220 μl. The solutions were mixed by briefly tapping the plate, and optical density at 405 nm was measured immediately (t = 0) and again after 20 min (t = 20 min) on a plate reader. The solutions were then used to calculate the GCase activity of the samples (U/l) based on the hydrolysis of 1 μM substrate/min by one unit of enzyme at pH 7.0.

### Western blot analysis

Western blot analysis was performed as previously described [44]. Briefly, protein samples (30 μg protein/lane) were separated by 12.5 % sodium dodecyl sulfate polyacrylamide gel electrophoresis, transferred to a polyvinylidene difluoride membrane, and then incubated with 3D5 mouse monoclonal antibody (1: 5000) overnight at 4°C. This was followed by incubation with horseradish peroxidase-conjugated goat anti-rabbit IgG (1: 5000; Vector Laboratories, Burlingame, CA, USA) for 1 h at room temperature. Immunoreactivity was detected using enhanced chemiluminescence reagent (Promega, Madison, WI, USA). β-Actin (1: 5000, Sigma–Aldrich, MO, USA) was used as a loading and internal control to enable sample normalization.

### Measurement of ceramide levels

Levels of ceramide in brain extracts were measured using a human ceramide ELISA kit (DRE12657; Rapidbio Biosource, West Hills, CA, USA) according to the instructions provided by the manufacturer.

### Measurement of PLK2 levels

Levels of PLK2 in brain extracts were measured using a human PLK2 ELISA kit (DRE12659; Rapidbio Biosource, West Hills, CA, USA) according to the manufacturer's instructions.

### Cell culture

MES23.5 dopaminergic cells (a gift from Dr. Weidong Le, Baylor College of Medicine) [45] were cultured in DMEM/F12 medium (Gibco, NY, USA) supplemented with 5% fetal calf serum and Sato's ingredients as described previously [46]. The cells were seeded on 96-well plates for cell viability assays. All the plates were pre-coated with poly-L-lysine as described previously.

### Cell viability assay

Cell viability was estimated using the MTT formazan colorimetric assay. 20 μL of MTT solution (5 mg/mL in PBS) was added to each well of 96-well plates and incubated in a humidified incubator with 5 % CO_2_ at 37°C for 4 h. The medium was removed, followed by addition of 100 μL of DMSO. The plates were centrifuged at 40000 rpm for 10 min. Optical density of the formazan product in solution was measured at 490 nm using a microplate reader (Mutiskan MK3, Thermo Scientific, USA).

### PI and Hoechst staining for detection of cell death

Cell nuclei were stained at 37°C for 10 min with Hoechst 33432 (Sigma–Aldrich, MO, USA, 0.5 μg/ml in PBS) and propidium iodide (PI, Sigma–Aldrich, MO, USA, 10 μg/ml in DMSO) [47]. The signals of PI and Hoechst 33432 were captured and analyzed by the GE IN Cell Analyzer 2000 High-Content Cellular Analysis System (GE Healthcare Bio-Sciences, Piscataway, NJ, USA) to evaluate the ratios of dead cells versus total cells.

### Statistical analysis

Data are expressed as the mean ± standard deviation (SD). The statistical analyses were performed using GraphPad Prism software 6.0 (GraphPad software, San Diego, California, USA). Data were analyzed by two-way ANOVA, followed by Tukey's multiple comparisons test to evaluate the differences between groups. *P* < 0.05 was considered statistically significant.

## References

[1] Rodriguez M, Rodriguez-Sabate C, Morales I, Sanchez A, Sabate M (2015). Parkinson's disease as a result of aging. Aging Cell.

[2] Hanson JC, Lippa CF (2009). Lewy body dementia. Int Rev Neurobiol.

[3] Jellinger KA (2003). Alpha-synuclein pathology in Parkinson's and Alzheimer's disease brain: incidence and topographic distribution--a pilot study. Acta Neuropathol.

[4] Jellinger KA (2009). Formation and development of Lewy pathology: a critical update. J Neurol.

[5] Baba M, Nakajo S, Tu PH, Tomita T, Nakaya K, Lee VM, Trojanowski JQ, Iwatsubo T (1998). Aggregation of alpha-synuclein in Lewy bodies of sporadic Parkinson's disease and dementia with Lewy bodies. Am J Pathol.

[6] Spillantini MG, Crowther RA, Jakes R, Hasegawa M, Goedert M (1998). Alpha-synuclein in filamentous inclusions of Lewy bodies from Parkinson's disease and dementia with lewy bodies. Proc Natl Acad Sci U S A.

[7] Colla E, Jensen PH, Pletnikova O, Troncoso JC, Glabe C, Lee MK (2012). Accumulation of toxic α-synuclein oligomer within endoplasmic reticulum occurs in alpha-synucleinopathy in vivo. J Neurosci.

[8] Malchiodi-Albedi F, Paradisi S, Matteucci A, Frank C, Diociaiuti M (2011). Amyloid oligomer neurotoxicity, calcium dysregulation, and lipid rafts. Int J Alzheimers Dis.

[9] Volles MJ, Lansbury PT (2003). Zeroing in on the pathogenic form of alpha-synuclein and its mechanism of neurotoxicity in Parkinson's disease. Biochemistry.

[10] Winner B, Jappelli R, Maji SK, Desplats PA, Boyer L, Aigner S, Hetzer C, Loher T, Vilar M, Campioni S, Tzitzilonis C, Soragni A, Jessberger S, Mira H, Consiglio A, Pham E, Masliah E, Gage FH, Riek R (2011). In vivo demonstration that alpha-synuclein oligomers are toxic. Proc Natl Acad Sci U S A.

[11] Outeiro TF, Putcha P, Tetzlaff JE, Spoelgen R, Koker M, Carvalho F, Hyman BT, McLean PJ (2008). Formation of toxic oligomeric alpha-synuclein species in living cells. PLoS One.

[12] Zhou W, Milder JB, Freed CR (2008). Transgenic mice overexpressing tyrosine-to-cysteine mutant human alpha-synuclein: a progressive neurodegenerative model of diffuse Lewy body disease. J Biol Chem.

[13] Cookson MR, van der Brug M (2008). Cell systems and the toxic mechanism(s) of alpha-synuclein. Exp Neurol.

[14] Martin ZS, Neugebauer V, Dineley KT, Kayed R, Zhang W, Reese LC, Taglialatela G (2012). α-Synuclein oligomers oppose long-term potentiation and impair memory through a calcineurin-dependent mechanism: relevance to human synucleopathic diseases. J Neurochem.

[15] Choi BK, Choi MG, Kim JY, Yang Y, Lai Y, Kweon DH, Lee NK, Shin YK (2013). Large alpha-synuclein oligomers inhibit neuronal SNARE-mediated vesicle docking. Proc Natl Acad Sci U S A.

[16] Kramer ML, Schulz-Schaeffer WJ (2007). Presynaptic alpha-synuclein aggregates, not Lewy bodies, cause neurodegeneration in dementia with Lewy bodies. J Neurosci.

[17] Schulz-Schaeffer WJ (2010). The synaptic pathology of alpha-synuclein aggregation in dementia with Lewy bodies, Parkinson's disease and Parkinson's disease dementia. Acta Neuropathol.

[18] Chen M, Wang T, Yue F, Li X, Wang P, Li Y, Chan P, Yu S (2015). Tea polyphenols alleviate motor impairments, dopaminergic neuronal injury, and cerebral α-synuclein aggregation in MPTP-intoxicated parkinsonian monkeys. Neuroscience.

[19] Zhou J, Broe M, Huang Y, Anderson JP, Gai WP, Milward EA, Porritt M, Howells D, Hughes AJ, Wang X, Halliday GM (2011). Changes in the solubility and phosphorylation of α-synuclein over the course of Parkinson's disease. Acta Neuropathol.

[20] Smith WW, Margolis RL, Li X, Troncoso JC, Lee MK, Dawson VL, Dawson TM, Iwatsubo T, Ross CA (2005). Alpha-synuclein phosphorylation enhances eosinophilic cytoplasmic inclusion formation in SH-SY5Y cells. J Neurosci.

[21] Arawaka S, Wada M, Goto S, Karube H, Sakamoto M, Ren CH, Koyama S, Nagasawa H, Kimura H, Kawanami T, Kurita K, Tajima K, Daimon M (2006). The role of G-protein-coupled receptor kinase 5 in pathogenesis of sporadic Parkinson's disease. J Neurosci.

[22] Kragh CL, Lund LB, Febbraro F, Hansen HD, Gai WP, El-Agnaf O, Richter-Landsberg C, Jensen PH (2009). Alpha-synuclein aggregation and Ser-129 phosphorylation-dependent cell death in oligodendroglial cells. J Biol Chem.

[23] Inglis KJ, Chereau D, Brigham EF, Chiou SS, Schobel S, Frigon NL, Yu M, Caccavello RJ, Nelson S, Motter R, Wright S, Chian D, Santiago P (2009). Polo-like kinase 2 (PLK2) phosphorylates alpha-synuclein at serine 129 in central nervous system. J Biol Chem.

[24] Bergeron M, Motter R, Tanaka P, Fauss D, Babcock M, Chiou SS, Nelson S, San Pablo F, Anderson JP (2014). In vivo modulation of polo-like kinases supports a key role for PLK2 in Ser129 α-synuclein phosphorylation in mouse brain. Neuroscience.

[25] Aubele DL, Hom RK, Adler M, Galemmo RA, Bowers S, Truong AP, Pan H, Beroza P, Neitz RJ, Yao N, Lin M, Tonn G, Zhang H (2013). Selective and brain-permeable polo-like kinase-2 (Plk-2) inhibitors that reduce α-synuclein phosphorylation in rat brain. Chem Med Chem.

[26] Mbefo MK, Paleologou KE, Boucharaba A, Oueslati A, Schell H, Fournier M, Olschewski D, Yin G, Zweckstetter M, Masliah E, Kahle PJ, Hirling H, Lashuel HA (2010). Phosphorylation of synucleins by members of the Polo-like kinase family. J Biol Chem.

[27] Wu J, Lou H, Alerte TN, Stachowski EK, Chen J, Singleton AB, Hamilton RL, Perez RG (2012). Lewy-like aggregation of α-synuclein reduces protein phosphatase 2A activity in vitro and in vivo. Neuroscience.

[28] Pérez-Revuelta BI, Hettich MM, Ciociaro A, Rotermund C, Kahle PJ, Krauss S, Di Monte DA (2014). Metformin lowers Ser-129 phosphorylated α-synuclein levels via mTOR-dependent protein phosphatase 2A activation. Cell Death Dis.

[29] Lee KW, Chen W, Junn E, Im JY, Grosso H, Sonsalla PK, Feng X, Ray N, Fernandez JR, Chao Y, Masliah E, Voronkov M, Braithwaite SP (2011). Enhanced phosphatase activity attenuates α-synucleinopathy in a mouse model. J Neurosci.

[30] Wu J, Lou H, Alerte TN, Stachowski EK, Chen J, Singleton AB, Hamilton RL, Perez RG (2012). Lewy-like aggregation of α-synuclein reduces protein phosphatase 2A activity in vitro and in vivo. Neuroscience.

[31] Grabowski GA (2008). Phenotype, diagnosis, and treatment of Gaucher's disease. Lancet.

[32] Sidransky E, Nalls MA, Aasly JO, Aharon-Peretz J, Annesi G, Barbosa ER, Bar-Shira A, Berg D, Bras J, Brice A, Chen CM, Clark LN, Condroyer C (2009). Multicenter analysis of glucocerebrosidase mutations in Parkinson's disease. N Engl J Med.

[33] Sidransky E, Lopez G (2012). The link between the GBA gene and parkinsonism. Lancet Neurol.

[34] Mazzulli JR, Xu YH, Sun Y, Knight AL, McLean PJ, Caldwell GA, Sidransky E, Grabowski GA, Krainc D (2011). Gaucher disease glucocerebrosidase and α-synuclein form a bidirectional pathogenic loop in synucleinopathies. Cell.

[35] Cooper AA, Gitler AD, Cashikar A, Haynes CM, Hill KJ, Bhullar B, Liu K, Xu K, Strathearn KE, Liu F, Cao S, Caldwell KA, Caldwell GA (2006). Alpha-synuclein blocks ER-Golgi traffic and Rab1 rescues neuron loss in Parkinson's models. Science.

[36] Thayanidhi N, Helm JR, Nycz DC, Bentley M, Liang Y, Hay JC (2010). Alpha-synuclein delays endoplasmic reticulum (ER)-to-Golgi transport in mammalian cells by antagonizing ER/Golgi SNAREs. Mol Biol Cell.

[37] Galadari S, Hago A, Patel M (2001). Effects of cations on ceramide-activated protein phosphatase 2A. Exp Mol Med.

[38] Murphy KE, Gysbers AM, Abbott SK, Tayebi N, Kim WS, Sidransky E, Cooper A, Garner B, Halliday GM (2014). Reduced glucocerebrosidase is associated with increased α-synuclein in sporadic Parkinson's disease. Brain.

[39] Braak H, Del Tredici, K Rüb U, de Vos RA, Jansen Steur EN, Braak E (2003). Staging of brain pathology related to sporadic Parkinson's disease. Neurobiol Aging.

[40] Yu S, Li X, Liu G, Han J, Zhang C, Li Y, Xu S, Liu C, Gao Y, Yang H, Uéda K, Chan P (2007). Extensive nuclear localization of alpha-synuclein in normal rat brain neurons revealed by a novel monoclonal antibody. Neuroscience.

[41] Sasakawa H, Sakata E, Yamaguchi Y, Masuda M, Mori T, Kurimoto E, Iguchi T, Hisanaga S, Iwatsubo T, Hasegawa M, Kato K (2007). Ultra-high field NMR studies of antibody binding and site-specific phosphorylation of -synuclein. Biochem Biophys Res Commun.

[42] Liu G, Chen M, Mi N, Yang W, Li X, Wang P, Yin N, Li Y, Yue F, Chan P, Yu S (2015). Increased oligomerization and phosphorylation of α-synuclein are associated with decreased activity of glucocerebrosidase and protein phosphatase 2A in aging monkey brains. Neurobiol Aging.

[43] Qi Z, Yang W, Liu Y, Cui T, Gao H, Duan C, Lu L, Zhao C, Zhao H, Yang H (2011). Loss of PINK1 function decreases PP2A activity and promotes autophagy in dopaminergic cells and a murine model. Neurochem Int.

[44] Alim MA, Hossain MS, Arima K, Takeda K, Izumiyama Y, Nakamura M, Kaji H, Shinoda T, Hisanaga S, Uéda K (2002). Tubulin seeds alpha-synuclein fibril formation. J Biol Chem.

[45] Crawford GD, Le WD, Smith RG, Xie WJ, Stefani E, Appel SH (1992). A novel N18TG29 mesencephalon cell hybrid expresses properties that suggest a dopaminergic cell line of substantia nigra origin. J Neurosci.

[46] Yu S, Zuo X, Li Y, Zhang C, Zhou M, Zhang YA, Uéda K, Chan P (2004). Inhibition of tyrosine hydroxylase expression in alpha-synuclein-transfected dopaminergic neuronal cells. Neurosci Lett.

[47] Liao TT, Jia RW, Shi YL, Jia JW, Wang L, Chua H (2011). Propidium iodide staining method for testing the cytotoxicity of 2,4,6-trichlorophenol and perfluorooctane sulfonate at low concentrations with Vero cells. J Environ Sci Health A Tox Hazard Subst Environ Eng.

